# Supplementary material for: A shared core microbiome in soda lakes separated by large distances
Source: Nat Commun. 2019 Sep 17;10:4230. doi: 10.1038/s41467-019-12195-5 (PMC6748926; doi:10.1038/s41467-019-12195-5)
Supplement: Supplementary file 1 — Supplementary Information [file 41467_2019_12195_MOESM1_ESM.pdf]

Supplementary Information

A shared core microbiome in soda lakes separated by large distances

Jackie K. Zorz[1§], Christine Sharp[1], Manuel Kleiner[2], Paul M.K. Gordon[3], Richard T. Pon[3], Xiaoli Dong[1], Marc Strous[1]

[1]Department of Geoscience, University of Calgary, Calgary, AB, T2N 1N4, Canada
[2]Department of Plant and Microbial Biology, North Carolina State University, Raleigh, North Carolina, 27695, USA
[3]Centre for Health Genomics and Informatics, University of Calgary, Calgary, AB, T2N 2T9, Canada

§corresponding author
Email address:
jacqueline.zorz@ucalgary.ca

List of Contents

Supplementary Tables

Supplementary Table 1 – Aqueous geochemistry of the four lakes
Supplementary Table 2 – List of 16S amplicon OTUs matched with 16S sequences from MAGs
Supplementary Table 3 – List of Gemmatimonadetes reference sequences for Figure 5 phylogenetic tree
Supplementary Table 4 – List of RuBisCO large subunit reference sequences for Figure 5 phylogenetic tree

Supplementary Figures

Supplementary Figure 1 – Expanded Gemmatimonadetes phylogenetic tree
Supplementary Figure 2 – Expanded RuBisCO phylogenetic tree of Gemmatimonadetes representatives

Supplementary Tables

Supplementary Table 1 – Aqueous geochemistry of the four lakes

Lake	Detection Limits	Deer	Deer	Probe	Probe	Good Enough	Good Enough	Last Chance	Last Chance
**Year**		**2015**	**2017**	**2015**	**2017**	**2015**	**2017**	**2015**	**2017**
**Latitude**		51.354 N	51.354 N	51.451 N	51.4508 N	51.330 N	51.3299 N	51.328 N	51.3276 N
**Longitude**		121.25 W	121.25 W	121.39 W	121.39 W	121.64 W	121.64 W	121.63 W	121.63 W
**Electrical Conductivity**		22.2	19.8	22.9	19.3	33.1	16.4	44.2	46.5
**B (mg L^{-1})**	0.01 mg L^{-1}	0.15	0.05	0.06	0.06	0.14	0.04	1.67	1.56
**Ba (mg L^{-1})**	0.0005 mg L^{-1}	0.17	0.02	0.02	0.02	0.03	0.01	0.04	0.05
**Ca (mg L^{-1})**	0.0005 mg L^{-1}	3.4	4.1	4.5	3.8	3.5	5	5	8.5
**Fe (mg L^{-1})**	0.005 mg L^{-1}	0.33	0.79	0.32	0.75	0.08	0.07	0.28	5.59
**K (mg L^{-1})**	0.1 mg L^{-1}	96.6	86.4	58	88.2	359.8	167	277.2	268.9
**Li (mg L^{-1})**	0.005 mg L^{-1}	0	0	0	0	0	0	0	0
**Mg (mg L^{-1})**	0.0005 mg L^{-1}	33.7	38	6.4	38.1	46.3	29.3	15.1	25.3
**Mn (mg L^{-1})**	0.001 mg L^{-1}	0.08	0.01	0	0.01	0.02	0	0	0.08
**Na (mg L^{-1})**	0.05 mg L^{-1}	7296	6576.2	7967	6575.3	11548.9	5053.7	17540	19733.9
**Si (mg L^{-1})**	0.0005 mg L^{-1}	1.6	4	1.5	3.6	1.4	1	5.3	16.4
**Sr (mg L^{-1})**	0.0005 mg L^{-1}	0.18	0.02	0.02	0.02	0.02	0.02	0.03	0.05
**pH**		10.1	10.4	10.1	10.4	10.1	10.7	10.1	10.3
**NH_3 (µM)**	0.5 µM	na	23.4	na	23.3	na	11	na	4.5
**Total Alkalinity (mM)**		252.02	274.47	254.28	275.04	355.51	208.57	443.94	631.45
**CO_3^{2-} (mM)**		57.01	111.72	59.3	112.11	86.11	87.3	109.52	274.13
**HCO_3^- (mM)**		195.01	51.03	194.98	50.82	269.4	33.97	334.42	83.2
**Cl^- (mM)**	0.07 µM	3.6	3.1	8.5	3.1	34.8	16.1	23	22.8
**SO_4^{2-} (mM)**	0.05 µM	0.22	0.26	0.48	0.21	13.8	7.01	27.72	31.31
**PO_4^{3-} (mM)**	0.16 µM	0	0	0.31	0.1	0.4	0.2	4.1	4.41
**NO_3^- (mM)**	0.16 µM	0	0	0	0	0	0	0	0

Supplementary Table 2 – List of 16S amplicon OTUs matched with 16S sequences from MAGs

OTU	*Bin*	*Identity (%)*
**OTU_792**	A1	96.571
**OTU_173**	A6	99.143
**OTU_85**	A7	100
**OTU_1**	A2	98.571
**OTU_34**	Ac2	100
**OTU_21**	Ac1	100
**OTU_77**	Ac4	100
**OTU_56**	B15	100
**OTU_78**	B9	100
**OTU_8**	B2	96.034
**OTU_647**	B1	99.143
**OTU_15**	B6	100
**OTU_2**	C1	100
**OTU_6**	C7	100
**OTU_3**	C5	100
**OTU_44**	C4	100
**OTU_25**	C6	100
**OTU_65**	C8	100
**OTU_527**	C9	97.714
**OTU_117**	Ch1	100
**OTU_116**	Ch2	100
**OTU_119**	My1	100
**OTU_48**	D1	100
**OTU_248**	De1	97.714
**OTU_7**	E1	100
**OTU_83**	F2	100
**OTU_246**	F1	99.714
**OTU_161**	G10	100
**OTU_11**	G2	100
**OTU_40**	G9	100
**OTU_33**	G7	100
**OTU_12**	G11	100
**OTU_203**	Ge2	100
**OTU_153**	Ge5	98.857
**OTU_13**	Ge1	100
**OTU_420**	F3	100
**OTU_240**	F4	100
**OTU_102**	P9	100
**OTU_178**	P4	100
**OTU_91**	P3	100
**OTU_226**	P5	100

OTU	*Bin*	*Identity (%)*
**OTU_149**	P6	100
**OTU_948**	P1	96.857
**OTU_360**	V1	98.286
**OTU_14**	S3	100
**OTU_75**	S2	99.714
**OTU_49**	S1	100
**OTU_96**	S4	98.857
**OTU_92**	S6	100

Supplementary Table 3 – List of Gemmatimonadetes reference sequences for Figure 5 phylogenetic tree.

Genbank accession (assembly)	cluster*	sample origin	Class	Order	Family	Genus
**GCA_000522985.1**	A	clinical	Gemmatimonadetes	Gemmatimonadales	Gemmatimonadaceae	Gemmatirosa
**GCA_001724275.1**	A	Cyanide and thiocyanate bioreactor	Gemmatimonadetes	Gemmatimonadales	Gemmatimonadaceae	SCN-70-22
**GCA_002215645.1**	A	soil (Laytonville, CA)	Gemmatimonadetes	Gemmatimonadales	Gemmatimonadaceae	2013-60CM-65-52
**GCA_002331565.1**	A	fresh water (Highgate Springs)	Gemmatimonadetes	Gemmatimonadales	Gemmatimonadaceae	Gemmatimonas
**GCA_002404335.1**	A	soil (Axel Heiberg Island, Canada, SRX710613)	Gemmatimonadetes	Gemmatimonadales	Gemmatimonadaceae	UBA4720
**GCA_002483225.1**	A	biofilm	Gemmatimonadetes	Gemmatimonadales	Gemmatimonadaceae	Gemmatimonas
**GCF_000010305.1**	A	activated sludge (phosphorus removal)	Gemmatimonadetes	Gemmatimonadales	Gemmatimonadaceae	Gemmatimonas
**GCF_000695095.2**	A	fresh water (Swan Lake, China)	Gemmatimonadetes	Gemmatimonadales	Gemmatimonadaceae	Gemmatimonas
**GCA_001802015.1**	B	Rifle well CD01 at time point 3 / C; 5m depth; 0.2 filter	Gemmatimonadetes	Gemmatimonadales	GWC2-71-9	GWC2-71-9
**GCA_001915025.1**	B	soil (Angelo meadow plot)	Gemmatimonadetes	Gemmatimonadales	GWC2-71-9	20CM-2-65-7
**GCA_001917625.1**	B	soil (Angelo meadow plot)	Gemmatimonadetes	Gemmatimonadales	GWC2-71-9	13-1-40CM-70-15
**GCA_001919115.1**	B	soil (Angelo meadow plot)	Gemmatimonadetes	Gemmatimonadales	GWC2-71-9	40CM-2-70-7
**GCA_001919865.1**	B	soil (Angelo meadow plot)	Gemmatimonadetes	Gemmatimonadales	GWC2-71-9	20CM-4-69-16
**GCA_001919895.1**	B	soil (Angelo meadow plot)	Gemmatimonadetes	Gemmatimonadales	GWC2-71-9	20CM-2-65-7
**GCA_001917665.1**	B*	soil (Angelo meadow plot)	Gemmatimonadetes	Gemmatimonadales	GWC2-71-9	20CM-4-69-16
**GCA_002291775.1**	C	seawater (ERX556000)	Gemmatimonadetes	SG8-23	UBA6960	UBA822
**GCA_002295725.1**	C	seawater (ERX552242)	Gemmatimonadetes	SG8-23	UBA6960	UBA822
**GCA_002313235.1**	C	seawater (ERX556139)	Gemmatimonadetes	SG8-23	UBA6960	UBA822
**GCA_002450735.1**	C	seawater (ERX552287)	Gemmatimonadetes	SG8-23	UBA6960	UBA6934
**GCA_002712565.1**	C	seawater (South Atlantic)	Gemmatimonadetes	SG8-23	UBA6960	UBA822
**GCA_002718595.1**	C	seawater (South Pacific)	Gemmatimonadetes	SG8-23	UBA6960	GCA-2718595
**GCA_002731735.1**	C	seawater (North Pacific Ocean)	Gemmatimonadetes	SG8-23	UBA6960	UBA822
**GCA_003447405.1**	C	seawater (ERR599072)	Gemmatimonadetes	SG8-23	UBA6960	UBA822

Genbank accession (assembly)	cluster*	sample origin	Class	Order	Family	Genus
**GCA_003482615.1**	C	seawater (ERR599115)	Gemmatimonadetes	SG8-23	UBA6960	UBA822
**GCA_003445355.1**	C	seawater	Gemmatimonadetes	SG8-23	UBA6960	UBA822
**GCA_002295045.1**	D	seawater (ERX555973)	Gemmatimonadetes	SG8-23	UBA6960	UBA887
**GCA_002311875.1**	D	seawater (hydrothermal vent)	Gemmatimonadetes	SG8-23	UBA6960	UBA887
**GCA_002313925.1**	D	seawater (ERX556139)	Gemmatimonadetes	SG8-23	UBA6960	UBA887
**GCA_002339385.1**	D	seawater (ERX556040)	Gemmatimonadetes	SG8-23	UBA6960	UBA2589
**GCA_002347035.1**	D	seawater (ERX555935)	Gemmatimonadetes	SG8-23	UBA6960	UBA2982
**GCA_002348265.1**	D	seawater (ERX555932)	Gemmatimonadetes	SG8-23	UBA6960	UBA2589
**GCA_002348465.1**	D	seawater (ERX555931)	Gemmatimonadetes	SG8-23	UBA6960	UBA2589
**GCA_002348665.1**	D	seawater (ERX555935)	Gemmatimonadetes	SG8-23	UBA6960	UBA887
**GCA_002390225.1**	D	seawater (North Sea subsurface)	Gemmatimonadetes	SG8-23	UBA6960	UBA2589
**GCA_002501085.1**	D	seawater (ERX555949)	Gemmatimonadetes	SG8-23	UBA6960	UBA887
**GCA_002724575.1**	D	seawater	Gemmatimonadetes	SG8-23	UBA6960	UBA887
**GCA_002730115.1**	D	seawater (North Pacific Ocean)	Gemmatimonadetes	SG8-23	UBA6960	UBA887
**GCA_003486145.1**	D	seawater (ERR599100)	Gemmatimonadetes	SG8-23	UBA6960	UBA2589
**GCA_003447875.1**	D	seawater (hydrothermal vent)	Gemmatimonadetes	SG8-23	UBA6960	UBA1138
**GCA_002346665.1**	Y	seawater (ERX555935)	Gemmatimonadetes	SG8-23	UBA6960	UBA2988
**GCA_002238605.1**		marine sponge (Aplysina aerophoba)	Gemmatimonadetes	SG8-23	BD2-11	BD2-11
**GCA_002238865.1**		marine sponge (Aplysina aerophoba)	Gemmatimonadetes	SG8-23	BD2-11	bin94
**GCA_001780825.1**		Rifle well FP-101 under low O2 conditions; 1.2 micron filter	Glassbacteria	GWA2-58-10	GWA2-58-10	GWA2-58-10
**GCA_002686955.1**		seawater (Arabian Sea)	GCA-2686955	GCA-2686955	GCA-2686955	GCA-2686955
**GCA_000012905.2**	outgroup	Rhodobacter sphaeroides	Alphaproteobacteria	Rhodobacterales	Rhodobacteraceae	Rhodobacter
**GCA_000017645.1**	outgroup	Xanthobacter autotrophicus	Alphaproteobacteria	Rhizobiales	Xanthobacteraceae	Xanthobacter
**GCA_000688515.1**	outgroup	Afifella pfennigii DSM 17143	Alphaproteobacteria	Rhizobiales	Rhodobiaceae	Afifella

* Too few alignment positions, not included in tree

Supplementary Table 4 – List of RuBisCO large subunit reference sequences for Figure 5 phylogenetic tree

Genbank accession	Class	Order	Family	Genus	Species/Isolate
**P27997**	Alphaproteobacteria	Rhodobacterales	Rhodobacteraceae	Rhodobacter	spaeroides
**P23011**	Alphaproteobacteria	Rhizobiales	Xanthobacteraceae	Xanthobacter	flavus
**P14959**	Ochrophyta: Raphidophyceae	Raphidophyceae incertae sedis	Raphidophyceae	Olisthodiscus	luteus
**P58348**	Alphaproteobacteria	Rhizobiales	Rhizobiaceae	Sinorhizobium	meliloti
**WP 051356539.1**	Alphaproteobacteria	Rhizobiales	Xanthobacteraceae	Azorhizobium	doebereinerae
**WP 037016557.1**	Alphaproteobacteria	Rhizobiales			YIM 77505
**WP 026380468.1**	Alphaproteobacteria	Rhizobiales	Rhodobiaceae	Afifella	pfennigii
**WP_011500311.1**	Methanomicrobia	Methanosarcinales	Methanosarcinaceae	Methanococcoides	burtonii DSM 6242
**WP_011385076.1**	Alphaproteobacteria	Rhodospirillales	Rhodospirillaceae	Magnetospirillum	magneticum AMB-1
**WP_011801368.1**	Betaproteobacteria	Burkholderiales	Comamonadaceae	Polaromonas	naphthalenivorans CJ2
**P04718**	Alphaproteobacteria	Rhodospirillales	Rhodospirillaceae	Rhodospirillum	rubrum
**AAG37859.1**	Dinophyceae	Suessiales	Symbiodiniaceae	Symbiodinium	sp. GN

Supplementary Figures

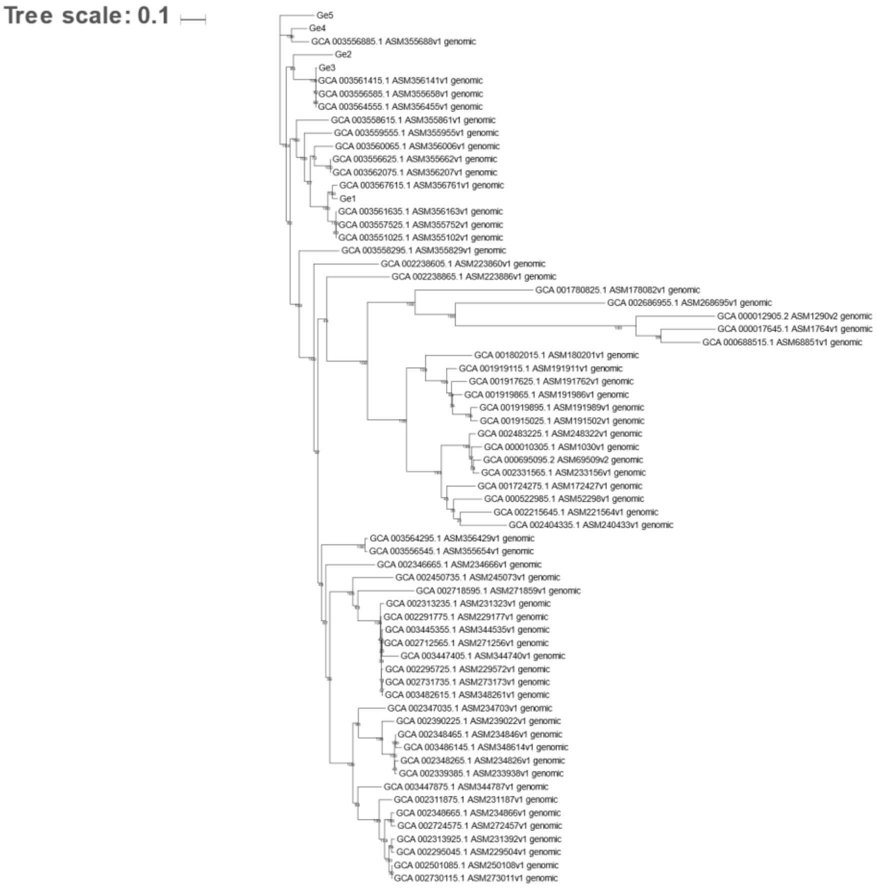

Supplementary Figure 1. Expanded Gemmatimonadetes phylogenetic tree. See also Figure 5a.

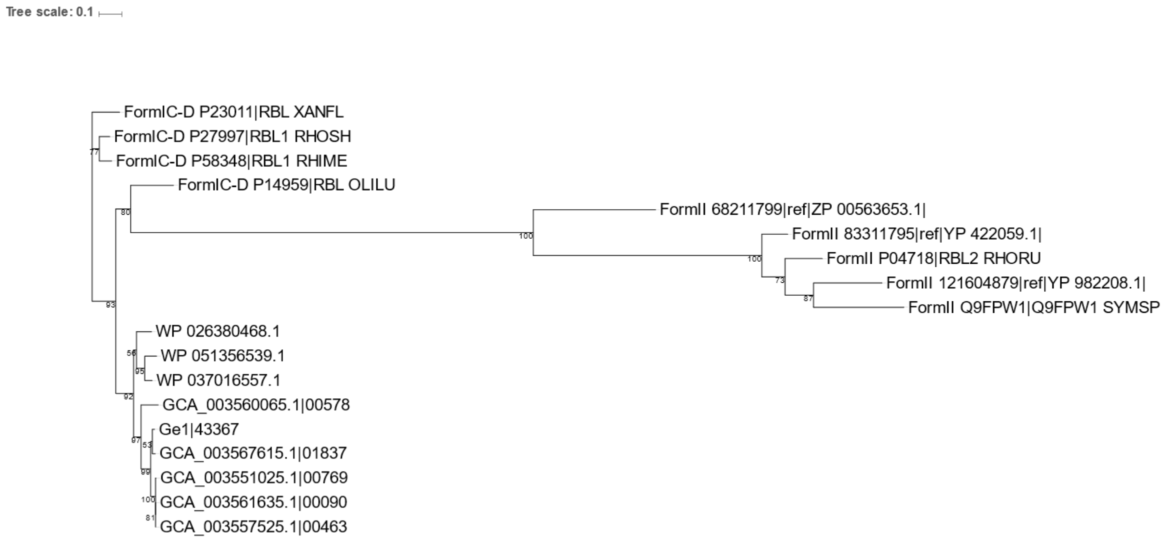

Supplementary Figure 2. Expanded RuBisCO phylogenetic tree of Gemmatimonadetes representatives. See also Figure 5b.
