# Supplementary material for: A shared core microbiome in soda lakes separated by large distances
Source: Nat Commun. 2019 Sep 17;10:4230. doi: 10.1038/s41467-019-12195-5 (PMC6748926; doi:10.1038/s41467-019-12195-5)
Supplement: Supplementary file 3 — Description of Additional Supplementary Files [file 41467_2019_12195_MOESM3_ESM.pdf]

Description of Additional Supplementary Files

File Name: Supplementary Data 1
Description: Operational Taxonomic Units, Bacterial 16S and 18S.

File Name: Supplementary Data 2
Description: Metagenome-assembled-genomes (MAGs) – GTDB classification, abundances, quality, relationships to Kulunda MAGs.

File Name: Supplementary Data 3
Description: Full length 16S rRNA gene sequences associated with MAGs.

File Name: Supplementary Data 4
Description: Co-occurrences of nearly identical variants of MAGs, showing no evidence for competitive exclusion.

File Name: Supplementary Data 5
Description: Evidence for diversifying evolution among some core genes of sets of MAG variants.

File Name: Supplementary Data 6
Description: Expression data for signature genes of different metabolic pathways (Figure 3).
