# Supplementary material for: A shared core microbiome in soda lakes separated by large distances
Source: Nat Commun. 2019 Sep 17;10:4230. doi: 10.1038/s41467-019-12195-5 (PMC6748926; doi:10.1038/s41467-019-12195-5)
Supplement: Supplementary file 10 — Reporting Summary [file 41467_2019_12195_MOESM10_ESM.pdf]

nature research

Corresponding author(s): Jackie K Zorz, Marc Strous

Last updated by author(s): Jul 29, 2019

Reporting Summary

Nature Research wishes to improve the reproducibility of the work that we publish. This form provides structure for consistency and transparency in reporting. For further information on Nature Research policies, see Authors & Referees and the Editorial Policy Checklist.

Statistics

For all statistical analyses, confirm that the following items are present in the figure legend, table legend, main text, or Methods section.

n/a	Confirmed	
☐	☒	The exact sample size (n) for each experimental group/condition, given as a discrete number and unit of measurement
☐	☒	A statement on whether measurements were taken from distinct samples or whether the same sample was measured repeatedly
☐	☒	The statistical test(s) used AND whether they are one- or two-sided<br>*Only common tests should be described solely by name; describe more complex techniques in the Methods section.*
☒	☐	A description of all covariates tested
☐	☒	A description of any assumptions or corrections, such as tests of normality and adjustment for multiple comparisons
☒	☐	A full description of the statistical parameters including central tendency (e.g. means) or other basic estimates (e.g. regression coefficient) AND variation (e.g. standard deviation) or associated estimates of uncertainty (e.g. confidence intervals)
☐	☒	For null hypothesis testing, the test statistic (e.g. F, t, r) with confidence intervals, effect sizes, degrees of freedom and P value noted<br>*Give P values as exact values whenever suitable.*
☒	☐	For Bayesian analysis, information on the choice of priors and Markov chain Monte Carlo settings
☒	☐	For hierarchical and complex designs, identification of the appropriate level for tests and full reporting of outcomes
☐	☒	Estimates of effect sizes (e.g. Cohen's d, Pearson's r), indicating how they were calculated

Our web collection on statistics for biologists contains articles on many of the points above.

Software and code

Policy information about availability of computer code

Data collection	Orion 960 Titrator<br>Varian 725-ES Inductively Coupled Plasma Optical Emission Spectrophotometer<br>Dionex ICS 2000 ion chromatograph<br>MiSeq benchtop sequencer<br>NextSeq 500 System<br>EASY-nLC 1000 Liquid Chromatograph<br>QExactive Plus hybrid quadrupole-Orbitrap mass spectrometer

Data analysis	EZ 960 software<br>MetaAmp Version 2.0<br>R version 3.5.2<br>vegan package 2.5-4<br>BBnorm (sourceforge.net/projects/bbmap)<br>BBMerge<br>MetaSpades version 3.10.0<br>BBMap<br>MetaBat<br>CheckM version 1.0.8<br>Phyloflash2<br>MetaErg (sourceforge.net/projects/metaerg/)<br>fastANI<br>MEGA7<br>GTDBtk (version 0.2.2, database release 86)<br>Proteome Discoverer version 2.0.0.802<br>Calis-P

For manuscripts utilizing custom algorithms or software that are central to the research but not yet described in published literature, software must be made available to editors/reviewers. We strongly encourage code deposition in a community repository (e.g. GitHub). See the Nature Research guidelines for submitting code & software for further information.

Data

Policy information about availability of data

All manuscripts must include a data availability statement. This statement should provide the following information, where applicable:

- Accession codes, unique identifiers, or web links for publicly available datasets
- A list of figures that have associated raw data
- A description of any restrictions on data availability

Amplicon sequences can be found under the Bioproject PRJNA377096. The 16S rRNA sequence Biosamples are: SAMN06456834, SAMN06456843, SAMN06456852, SAMN06456861, SAMN09986741-SAMN09986751, and the 18S rRNA sequence Biosamples are: SAMN09991649-SAMN09991660. The metagenome raw reads and metagenome assembled genomes can also be found under the Bioproject PRJNA377096. The Biosamples for the metagenome raw reads are SAMN10093821-SAMN10093824, and the Biosamples for the MAGs are SAMN10237340-SAMN10237430. The metaproteomics data has been deposited to the ProteomeXchange Consortium via the PRIDE partner repository with the dataset identifier PXD011230.

Field-specific reporting

Please select the one below that is the best fit for your research. If you are not sure, read the appropriate sections before making your selection.

☐ Life sciences ☐ Behavioural & social sciences ☒ Ecological, evolutionary & environmental sciences

For a reference copy of the document with all sections, see nature.com/documents/nr-reporting-summary-flat.pdf

Ecological, evolutionary & environmental sciences study design

All studies must disclose on these points even when the disclosure is negative.

Study description	In this study we used amplicon sequencing, metaproteomes, and metagenomes to address fundamental questions on the microbial ecology of soda lake mats. Samples were taken from highly productive benthic microbial mats from four lakes in the interior of British Columbia Canada that had high pH (>10), and were highly alkaline. We further explored the concept of microbial biogeography by comparing the metagenome-assembled-genomes from the present study in western Canada, to the metagenome-assembled-genomes from a recently published study of soda lakes in Siberia.
Research sample	The benthic microbial mats from four highly alkaline soda lakes were chosen for study in this experiment. These mats are of interest due to their high productivity, and also due to their ability to thrive in extremophilic (high alkalinity, high pH) conditions. The recently published study of Siberian soda lakes was chosen for comparison with our data, because it is the only other existing dataset in the literature that targets soda lake systems and contains high quality metagenome-assembled-genomes.
Sampling strategy	Benthic microbial mats were collected using a small bucket. Mats were immediately frozen in sterile tubes, transported on dry ice, and stored at -80°C within 2 days of sampling. In 2015 and 2017, water samples for aqueous geochemistry were also taken, transported on dry ice, and stored at -80°C until analysis. Transport of samples from the field to the lab on dry ice took less than 48 hours.
Data collection	CS and JZ collected the samples in the field, extracted DNA, and conducted amplicon sequencing. PG and RP performed metagenome sequencing of select samples. MK perfomed metaproteomics analysis using the Orbitrap mass spectrometer. MS, XD, and JZ analyzed metagenome and metaproteome data. MS performed the analytical comparison of the present metagenome data to the Siberian metagenomes.
Timing and spatial scale	Sampling from benthic microbial mats was conducted at the Cariboo Plateau soda lakes each May for four years (2014-2017). The lakes freeze in winter and occasionally dry up in summer, so May was chosen as an intermediate time to sample to ensure that the

lakes were still liquid. Deer Lake was dry in 2016, and thus no sample was taken from the lake that year.

Data exclusions

No data was excluded from the manuscript

Reproducibility

Amplicon data from the same lake sampled over different years were consistently similar, showing that the sequencing and analysis methods, as well as the microbial communities were consistent between years. Technical replicates were conducted for both amplicon sequencing and metaproteomics analysis.

Randomization

Samples were allocated into groups based on the lake they were sampled from, and for amplicon sequencing, also based on the year of sampling. For metaproteomics analysis, technical replicate samples were analyzed in a random order by the mass spectrometer.

Blinding

Our study focuses on microbial community assembly and biogeography, and thus blinding was not required in our experimental design.

Did the study involve field work? ☒ Yes ☐ No

Field work, collection and transport

Field conditions

Samples were taken in May, where temperature commonly ranged between 15 and 20 degrees C.

Location

Deer Lake: 51.354 N 121.25 W
Goodenough Lake 51.330 N 121.64 W
Last Chance Lake: 51.451 N 121.39 W
Probe Lake: 51.451 N 121.25 W
All samples were taken from benthic microbial mats, and lakes were approximately 1 m deep

Access and import/export

The lakes in the Cariboo Plateau are outside of national or provincial parks and are thus accessible to everyone. Lakes are reachable by country roads, and sampling could be carried out with minimal environmental affect.

Disturbance

Disturbance was minimal as only small volumes of samples were taken, and nothing was left at the sampling site

Reporting for specific materials, systems and methods

We require information from authors about some types of materials, experimental systems and methods used in many studies. Here, indicate whether each material, system or method listed is relevant to your study. If you are not sure if a list item applies to your research, read the appropriate section before selecting a response.

Materials & experimental systems

n/a	Involved in the study	
☒	☐	Antibodies
☒	☐	Eukaryotic cell lines
☒	☐	Palaeontology
☒	☐	Animals and other organisms
☒	☐	Human research participants
☒	☐	Clinical data

Methods

n/a	Involved in the study	
☒	☐	ChIP-seq
☒	☐	Flow cytometry
☒	☐	MRI-based neuroimaging
